# Stable 293 T and CHO cell lines expressing cleaved, stable HIV-1 envelope glycoprotein trimers for structural and vaccine studies

**DOI:** 10.1186/1742-4690-11-33

**Published:** 2014-04-25

**Authors:** Nancy PY Chung, Katie Matthews, Helen J Kim, Thomas J Ketas, Michael Golabek, Kevin de los Reyes, Jacob Korzun, Anila Yasmeen, Rogier W Sanders, Per Johan Klasse, Ian A Wilson, Andrew B Ward, Andre J Marozsan, John P Moore, Albert Cupo

**Affiliations:** 1 Department of Microbiology and Immunology, Weill Cornell Medical College, New York, NY, USA; 2 Department of Integrative Structural and Computational Biology, IAVI-Neutralizing Antibody Center, CHAVI-ID, The Scripps Research Institute, La Jolla, California, USA; 3 The Skaggs Institute for Chemical Biology, The Scripps Research Institute, La Jolla, California, USA; 4 Department of Medical Microbiology, Academic Medical Center, University of Amsterdam, Amsterdam, The Netherlands; 5 Current address: Alexion Pharmaceuticals, Cheshire, Connecticut, USA; 6 Current address: Innovimmune Biotherapeutics, Inc, New York, NY, USA

**Keywords:** HIV-1 envelope glycoproteins, BG505 SOSIP.664 gp140 trimers, Neutralizing antibodies, Vaccination, Flp-In™ cells

## Abstract

**Background:**

Recombinant soluble, cleaved HIV-1 envelope glycoprotein SOSIP.664 gp140 trimers based on the subtype A BG505 sequence are being studied structurally and tested as immunogens in animals. For these trimers to become a vaccine candidate for human trials, they would need to be made in appropriate amounts at an acceptable quality. Accomplishing such tasks by transient transfection is likely to be challenging. The traditional way to express recombinant proteins in large amounts is via a permanent cell line, usually of mammalian origin. Making cell lines that produce BG505 SOSIP.664 trimers requires the co-expression of the Furin protease to ensure that the cleavage site between the gp120 and gp41 subunits is fully utilized.

**Results:**

We designed a vector capable of expressing Env and Furin, and used it to create Stable 293 T and CHO Flp-In™ cell lines through site-specific recombination. Both lines produce high quality, cleaved trimers at yields of up to 12–15 mg per 1 × 10^9^ cells. Trimer expression at such levels was maintained for up to 30 days (10 passages) after initial seeding and was consistently superior to what could be achieved by transient transfection. Electron microscopy studies confirm that the purified trimers have the same native-like appearance as those derived by transient transfection and used to generate high-resolution structures. They also have appropriate antigenic properties, including the presentation of the quaternary epitope for the broadly neutralizing antibody PGT145.

**Conclusions:**

The BG505 SOSIP.664 trimer-expressing cell lines yield proteins of an appropriate quality for structural studies and animal immunogenicity experiments. The methodology is suitable for making similar lines under Good Manufacturing Practice conditions, to produce trimers for human clinical trials. Moreover, any *env* gene can be incorporated into this vector system, allowing the manufacture of SOSIP trimers from multiple genotypes, either by transient transfection or from stable cell lines.

## Background

The development of a vaccine against HIV-1 infection remains a significant global health problem. One of the major vaccine design concepts is the induction of high titer broadly neutralizing antibodies (bNAbs) that are capable of preventing HIV-1 entry into its target cells, and hence intervening against virus transmission [1]. The only relevant target for bNAbs is the Env complex, or spike, on the virus surface [2,3]. This complex, a trimer of gp120/gp41 heterodimers, attaches to receptors on target cells and then mediates fusion of the viral and cell membranes [4]. Any antibodies that can bind to a sufficient number of functional Env spikes will impede one or more stages in the receptor-binding and fusion process, thereby neutralizing infectivity [2].

The induction of bNAbs by vaccination remains elusive. Among relevant strategies is the creation of structural and antigenic mimics of the native, virion-associated Env spike. To do so involves inserting a stop codon to truncate the gp140 prior to the membrane-spanning domain, thereby yielding soluble proteins that can be secreted from producer cells and purified. Unfortunately, without suitable sequence modifications, soluble gp140s are highly unstable and either disintegrate completely into their constituents or adopt non-native configurations that may be compromised from the perspective of bNAb induction [5,6]. One effective strategy for making soluble, native-like trimers involves introducing a disulfide bond (designated SOS) to link the gp120 and gp41 ectodomain (gp41_ECTO_) components, combined with a point substitution (I559P) in gp41_ECTO_ that creates additional stability [7-9]. Proteolytic cleavage between the gp120 and gp41 subunits is also critical for making native-like trimers, and is facilitated by optimizing the cleavage site and co-expressing the Furin protease [8,9]. At present, the most advanced soluble, cleaved trimers, based on a subtype A founder virus, are designated BG505 SOSIP.664 [10-12]. These highly homogeneous trimers closely resemble virus spikes when visualized by negative stain electron microscopy (EM) [10]. They also have highly favorable antigenic properties, expressing multiple bNAb epitopes while occluding most epitopes for non-neutralizing antibodies (non-NAbs) [6,10]. In addition, the structure of these trimers has been determined by both x-ray diffraction and cryo-EM at ~5-6 Å resolution [13,14]. Animal immunogenicity studies are in progress.

To facilitate the production of BG505 SOSIP.664 gp140 trimers as pre-clinical research reagents, and to establish a proof-of-concept for their future manufacture as clinical-grade immunogens, we have assessed whether they can be expressed in permanent cell lines. A complexity in the cell line design strategy was the need to co-express Furin, to ensure the trimers are fully cleaved. Another important requirement for a stable mammalian cell line is a defined integration site(s). The Flp-In™ system seemed an appropriate choice; it has, for example, been used to produce influenza hemagglutinin-based vaccines [15]. Here, we show that the Flp-In™ method can be used to make both 293 T and CHO cell lines that yield substantial amounts (up to 12–15 mg from 1 × 10^9^ cells) of high quality trimers. The purified trimers are fully cleaved, have appropriate antigenic properties and, when viewed by negative stain EM, appear identical to ones produced by transient transfection [6,10]. The method used to make the lines is speedy and flexible, which will facilitate the future production of additional SOSIP trimers of improved designs and/or different genotypes. It should also be adaptable to Good Manufacturing Practice (GMP) conditions appropriate for making immunogens for human vaccine trials.

## Results

### Design of pAM/C BG505 vector expressing BG505 SOSIP.664 gp140 and Furin

**Figure 1:**
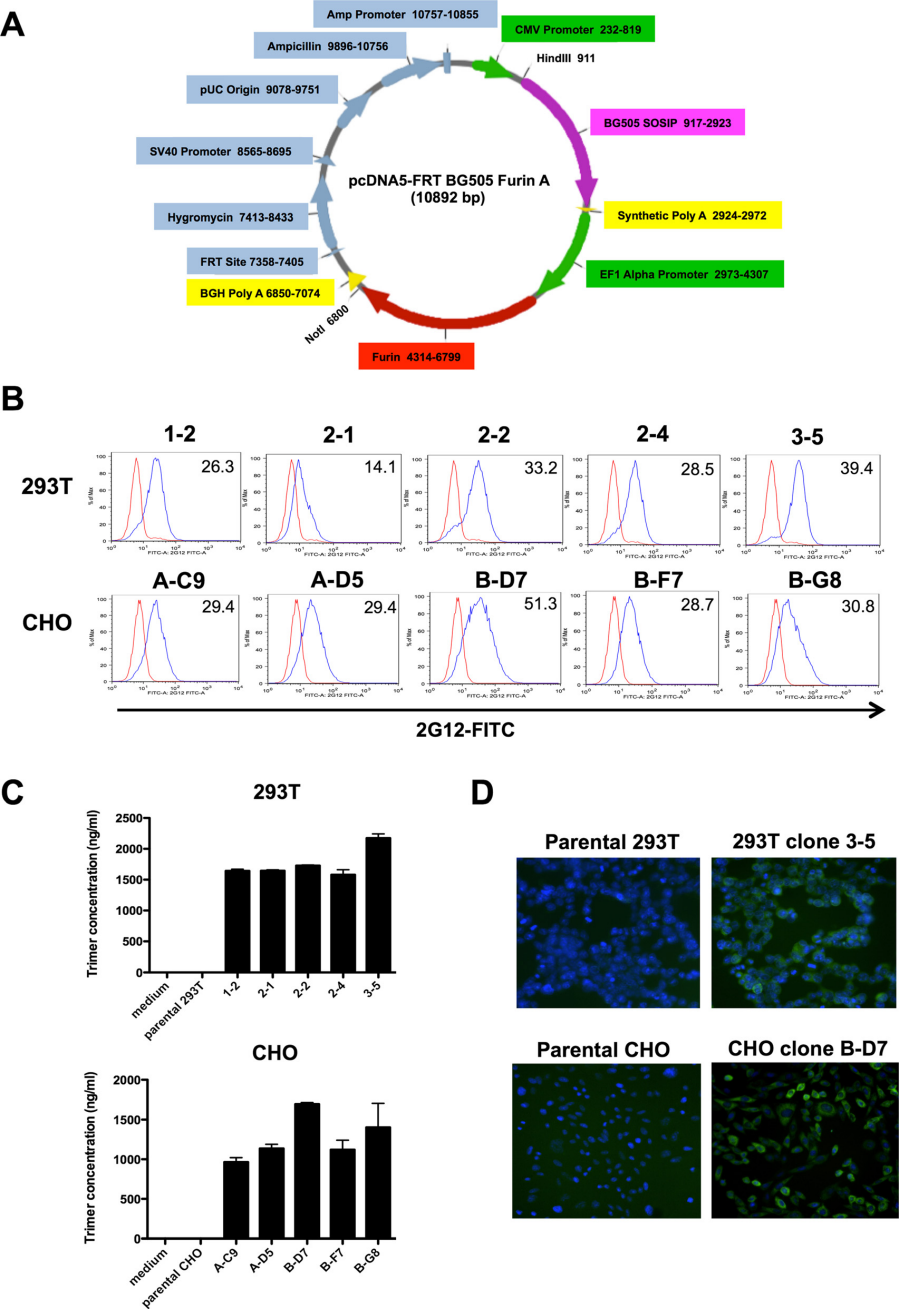
**Vector for constitutive secretion of BG505 SOSIP.664 gp140 in a Flp-In™ based expression system, and stable cell line selection. (A)** Design of the pAM/C construct for expressing BG505 SOSIP.664 gp140. The plasmid map shows the site of the *env* and *furin* gene insertions, the promoters and the Poly A sequences. **(B)** Intracellular Env expression in transfected 293 T and CHO cells. The histograms represent parental cells (red) and stable cell clones (blue); the numbers (top right of each panel) are the mean fluorescence intensity (MFI) values after staining with FITC-2G12. **(C)** Secretion of BG505 SOSIP.664 gp140 trimers by Stable 293 T and CHO cell clones. The trimer concentrations in the culture supernatants were determined by ELISA using 2G12 and bio-PGT145. **(D)** Fluorescent microscopy of stable cell clones. Cells were grown in an 8-well chamber slide, treated with Brefeldin A, fixed, permeabilized and stained for Env (FITC-2G12; green) or nuclear DNA (DAPI; blue). The left panels show parental 293 T and CHO cells, the right, the stable cell clones.

The pcDNA5/FRT vector contains multiple promoters and polyadenylation sites [16]. The choice of elements to use in the pAM/C BG505 vector was determined by assessing the priority of expression. Thus, we elected to use the highest activity (CMV) promoter for Env expression (to maximize trimer production), but a lower activity promoter (EFI Alpha) for Furin (to reduce the risk of cytotoxicity). When choosing the promoters and polyadenylation sites, we also considered the elements already present within the pcDNA5/FRT vector. For example, we could not use the SV40 promoter and Poly A for expressing Env or Furin because these elements are already active in the vector. Overall, we decided that the most efficient additions to the pcDNA5/FRT vector would be the EF1 Alpha promoter and a synthetic Poly A from New England BioLabs (modeled from the HSV Thymidine Kinase Poly A), between the *HindIII* and *NotI* cloning sites (Figure 1A).

A Flp Recombination Target (FRT) site in the pcDNA5/FRT vector is linked to the hygromycin-resistance gene, which allows for Flp recombinase-mediated integration and the selection of a stable cell line. The complete BG505 SOSIP.664 gp140 sequence was cloned into pcDNA5/FRT between the *HindIII* and *NotI* sites, under the control of the CMV promoter to promote high-level constitutive Env expression (Figure 1A). Since complete cleavage of Env at the gp120-gp41_ECTO_ juncture is essential for the production of native-like trimers [5,6,9,17] we also inserted the *furin* gene, in this case under the control of the weaker EFI Alpha promoter. The resulting plasmid that contains both the BG505 SOSIP.664 gp140 and Furin sequences is designated pAM/C BG505 (Figure 1A).

### Selection and propagation of Stable 293 T and CHO cell lines expressing BG505 SOSIP.664 gp140

The pAM/C BG505 vector was co-transfected with vector pOG44, which encodes the Flp recombinase that mediates integration of the pcDNA5/FRT vector into the FRT site of Flp-In™ cells. Using the Flp-In™ system, we obtained four potentially stable preliminary cell lines, 293 T lines 13 and 15 and CHO lines A and B. To eliminate the possibility that these initial lines were non-isogenic (i.e., genetically mixed), we next performed limiting dilution on the 293 T Flp-In™ line 13 and the CHO lines A and B, as these three consistently expressed the highest Env levels judged by dot blot using MAb 2G12. Limiting dilution resulted in 32 potential 293 T cell clones and 10 potential CHO cell clones. We used FITC-labeled MAb 2G12 (FITC-2G12) and FACS to assess Env expression and clonality; this procedure identified 293 T clone 13 #3-5 and CHO clone B-D7 as the highest-expressing clones for further propagation (Figure 1B and data not shown). An ELISA based on 2G12 capture of Env proteins followed by detection of trimers with biotinylated MAb PGT145 (bio-PGT145) confirmed that culture supernatants from these clones contained the highest quantities of trimers: 2.1 μg/ml for 293 T clone 13 #3-5 and 1.7 μg/ml for CHO clone B-D7 (Figure 1C). Staining with FITC-2G12 in the presence of Brefeldin A showed that Env proteins accumulated within the cell for both these clones, but were absent from the parental controls (Figure 1D).

### Sustained intracellular Env expression in Stable 293 T and CHO cell lines

**Figure 2:**
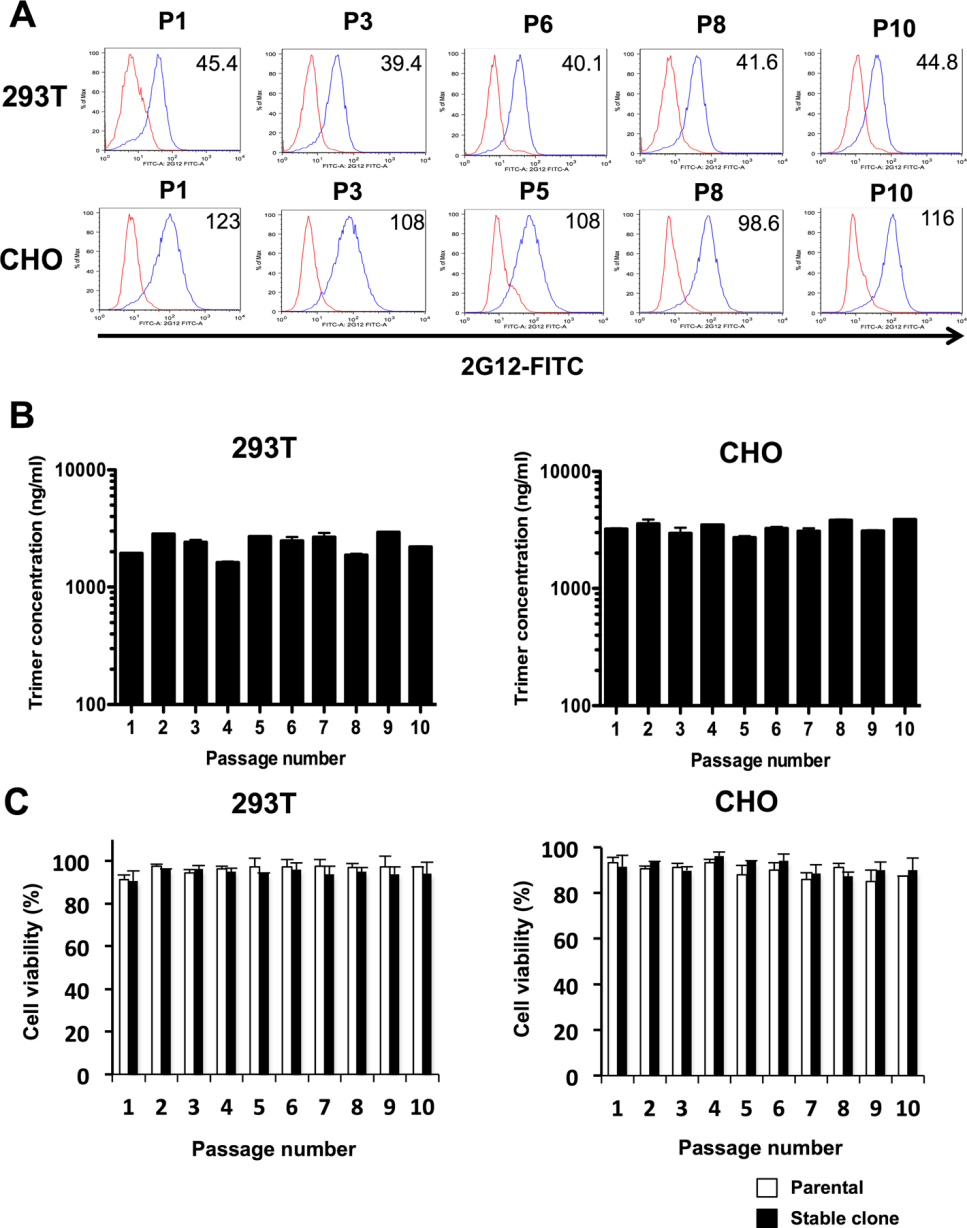
**Sustained expression of BG505 SOSIP.664 gp140 by stable cell lines. (A)** Intracellular Env expression with continued passage of the 293 T 13# 3–5 and CHO B-D7 stable cell lines (blue curves). The fixed and permeabilized cells were stained with FITC-2G12 (20 μg/ml) after culture for 6 h in the presence of Brefeldin A. MFI values for the Env-expressing clones are recorded in the top right corner of each histogram. Parental cells served as negative controls (red curves show MFI values: ranges 9.12-12.9 for 293 T cells and 7.23-13.2 for CHO cells). **(B)** Production of BG505 SOSIP.664 gp140 trimers by the stable cell lines throughout the culture period, as determined by ELISA using 2G12 and bio-PGT145. **(C)** Viability of stable cell clones during passage. The cells were stained with trypan blue, with the percentage of viable cells (parental *vs.* stable) shown as the passage number increases.

After initial seeding, approximately constant levels of intracellular Env were detected during ten subsequent passages (P1-10, one passage every 4 days) of the 293 T clone 13 #3-5 and the CHO clone B-D7. Both lines are therefore stable and not prone to genetic instability (Figure 2A).

The production of BG505 SOSIP.664 gp140 trimers, as judged by ELISA, was also steady over time during passages 1 through 10, with yields in the range of 2 and 3 μg/ml of supernatant for the 293 T and CHO cell clones, respectively (Figure 2B). Cell viability (parental *vs.* stable cell lines) was 91-98% *vs.* 91-96% for 293 T, and 86-95% *vs.* 87-94% for CHO, throughout the culture period, implying that neither Env nor Furin was cytotoxic to these lines (Figure 2C). Overall, the two stable cell lines could be passaged for 5 weeks without any observable reduction in Env expression or cell viability. The BG505 SOSIP.664 trimers can, therefore, be produced by the same batch of cells for at least this period after initial seeding, and probably far longer.

### Biochemical characterization of BG505 SOSIP.664 trimers from 293 T and CHO stable cell lines

**Figure 3:**
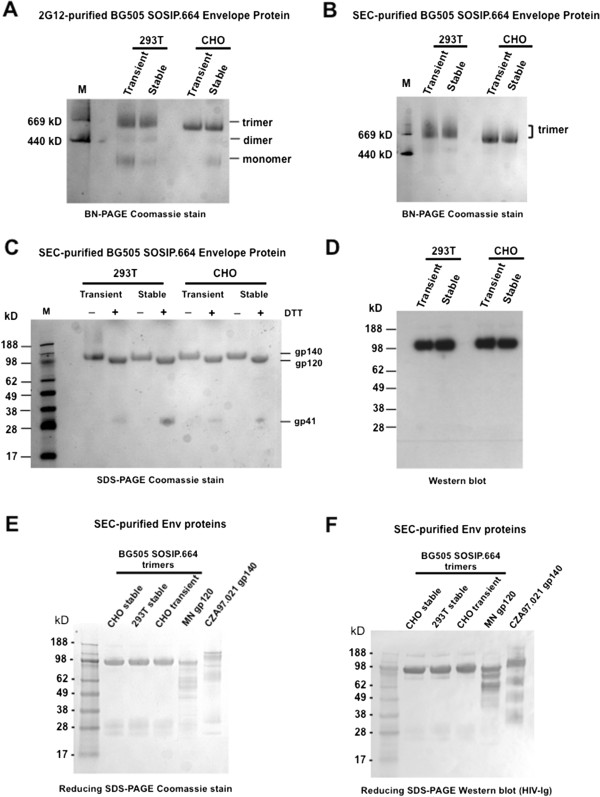
**Biochemical characterization of cell line-derived BG505 SOSIP.664 gp140 trimers and comparator proteins. (A)** BN-PAGE analysis of 2G12-purified Env proteins produced by the BG505 SOSIP.664-expressing 293 T and CHO stable cell lines. The gels were stained with Coomassie blue. The molecular weights of marker (M) proteins (thyroglobulin, 669 kDa and ferritin, 440 kDa) are indicated. **(B)** A similar BN-PAGE analysis, but of SEC-purified trimers. **(C)** SDS-PAGE analysis of SEC-purified BG505 SOSIP.664 trimers, under non-reducing (- DTT) and reducing (+ DTT) conditions, followed by Coomassie blue staining. Cleavage of gp120 from gp41_ECTO_ is assessed by the conversion of the gp140 band to gp120 in the presence of DTT; the released gp41_ECTO_ subunit is not always stained strongly. **(D)** Western blot analysis of a reducing SDS-PAGE gel to assess the quality of SEC-purified BG505 SOSIP.664 trimers. The blots were probed with the anti-gp120 MAb, ARP 3119. No Env degradation products arising from V3-clipping or other proteolysis events are visible. **(E)** Reducing SDS-PAGE analysis of SEC-purified BG505 SOSIP.664 trimers and comparator Env proteins, followed by Coomassie blue staining. The comparator proteins were also SEC-purified to yield the 120-kDa fraction for MN gp120 and the “trimer” fraction (i.e., proteins containing three gp120 and three gp41_ECTO_ subunits) for uncleaved CZA97.012 gp140. Multiple degradation and/or aggregation products derived from MN gp120 and CZA97.012 gp140 are visible, but none from the BG505 SOSIP.664 trimers. **(F)** Western blot analysis of the same gel shown in **(E)**. The detection antibodies were a pool (1:1:1) of polyclonal HIV-Igs derived from subtype A, B and C infections. The various aggregates or degradation products seen in **(E)** are Env-based.

We next assessed the quantity and quality of the trimers produced by the stable cell lines, in comparison with transiently transfected 293 T and CHO cells. In all cases, the trimers were purified by 2G12-affinity chromatography followed by size exclusion chromatography (SEC) [6,10]. A Coomassie blue-stained native (BN-PAGE) gel showed that the 2G12-enriched Env proteins were mostly trimeric, with only modest amounts of dimers and/or monomers visible (Figure 3A). The trimer fractions were then purified by SEC. Gel-electrophoretic analyses under native conditions (BN-PAGE, followed by Coomassie blue staining) showed that the purities of the different trimer preparations (cell lines *vs.* transient transfection) were indistinguishable (Figure 3B). The CHO cell-derived trimers migrated slightly more quickly than their 293 T counterparts, probably reflecting subtle, cell-dependent differences in glycan profiles that affect mobility during electrophoresis (Figure 3B).

The yield of purified trimers from the 293 T stable cell line was 12 mg (range 10–15 mg) per 1 × 10^9^ cells, which is 10-fold greater than when the same number of 293 T cells were transiently transfected (range 1.25-1.5 mg) (Table 1). For the stable CHO cell line *vs.* transiently transfected CHO cells, the corresponding values were 12 mg (range 10–15 mg) and 0.375 mg (range 0.25-0.5 mg), a ~32-fold differential.

**Table 1:** Production of BG505 SOSIP.664 gp140 trimers by stable cell lines relative to transiently transfected cells

**Production system**	**Purified trimer yield per 1 × 10**^**9 **^**cells**
CHO PEI transfection	0.25-0.5 mg
**CHO stable cell line**	**10-15 mg**
293 T PEI transfection	1.25-1.5 mg
**293 T stable cell line**	**10-15 mg**

The various SEC-purified BG505 SOSIP.664 trimer preparations were fully cleaved. Thus, when the reducing agent dithiothreitol (DTT) was included in SDS-PAGE gels, the gp140 proteins dissociated into their gp120 and gp41_ECTO_ subunits (Figure 3C). Note that the gp41_ECTO_ fragments stain poorly and are not always visible.

We next analyzed the purified trimers on a reducing SDS-PAGE gel followed by western blotting, with the goal of identifying whether any proteolytic degradation events occur during production or purification (Figure 3D). The detection MAb, ARP 3119, binds to a well-conserved, linear epitope within the N-terminal half of gp120. In other studies, we confirmed that it recognizes the 70-kDa (but not the 50-kDa) fragment that is produced when monomeric MN gp120 is clipped by proteases within the V3 region (data not shown). When the BG505 SOSIP.664 trimer blots were probed with ARP 3119, only a 120-kDa band (i.e., gp120) was detected, with no degradation products visible (Figure 3D). To seek any Env fragments that might escape detection by ARP 3119, we performed a similar reducing SDS-PAGE and Western blotting analysis but probed the blots with polyclonal HIV-Ig (Figures 3E and F). As positive controls, we included two other types of Env protein that are known to be vulnerable to proteolytic degradation, i.e., a monomeric gp120 and an uncleaved gp140 that contains three gp120 and three gp41_ECTO_ subunits [5,6,18,19]. The BG505 SOSIP.664 trimers are from subtype A, the gp120 protein, MN, is from subtype B, and the uncleaved gp140, CZA97.012, from subtype C [20]. Accordingly, we used a HIV-Ig pool based on sera from different individuals infected with viruses from all three subtypes. The comparator gp120 and gp140 proteins were SEC-purified and each migrated as a single band of appropriate size when analyzed by BN-PAGE and Coomassie blue staining (data not shown).

The Coomassie blue-stained, reducing SDS-PAGE gel again revealed no degradation products of the BG505 SOSIP.664 trimers; the major band corresponds to the gp120 subunit and a less well stained, smaller band is the gp41_ECTO_ moiety. In contrast, multiple degradation products were clearly visible in the SEC-purified monomeric gp120 and uncleaved gp140 preparations. The dominant bands from the gp120 monomers were the characteristic 70 kDa and 50 kDa fragments arising from V3 clipping. However, additional, unknown fragments of the uncleaved gp140 were also present, as well as high m.wt. bands corresponding to proteins that spontaneously aggregate after SEC-purification (Figure 3E). These observations were reinforced by Western blots, which confirmed that the various additional gp120- or gp140-derived bands were Env proteins. Again, however, there were no signs of degradation or aggregation with the various cell sources of BG505 SOSIP.664 trimers (Figure 3F).

### Surface plasmon resonance and ELISA analysis of BG505 SOSIP.664 gp140 trimers produced from Stable 293 T and CHO cell lines

The trimers produced by the permanent cell lines do not contain D7324-epitope or His-tags, which precludes assessment of their antigenicity by the SPR and capture ELISA methods for tagged trimers that we have previously described [6,10]. We therefore devised SPR and ELISA methods suitable for use with non-tagged trimers derived from both the permanent 293 T and CHO cell lines and, for comparison, made by transient transfection.

**Figure 4:**
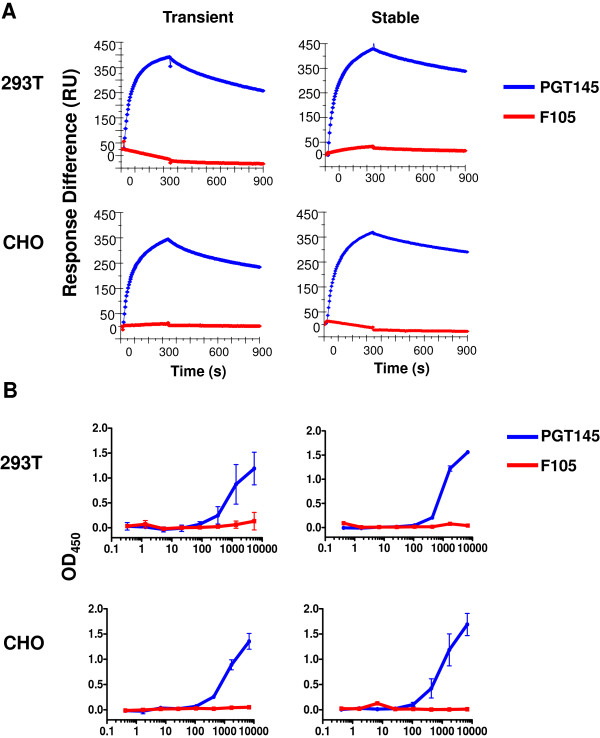
**Antigenicity of cell line-derived BG505 SOSIP.664 gp140 trimers. (A)** SPR sensorgrams for the PGT145 bNAb and the F105 non-NAb. Each MAb was captured onto the chip by an immobilized anti-Fc Ab, and the binding of BG505 SOSIP.664 gp140 trimers (200 nM) from the various cell sources was recorded as response difference (RU) after background correction: PGT145 bound strongly and similarly to the trimers of different origin, but F105 binding was undetectable. Each curve represents one of two similar replicates. **(B)** Representative binding curves in the 2G12-capture ELISA for the same PGT145 bNAb and F105 non-NAb to BG505 SOSIP.664 gp140 trimers from the various cell sources. The plotted OD values have been background corrected (i.e., with no gp120 present). Similar data were obtained in three experiments of the same design.

For SPR, we immobilized anti-Env MAbs on CM5 chips and flowed the purified BG505 SOSIP.664 trimers over them, recording binding as the response difference (RU). Immobilized bNAb PGT145, to a quaternary structure-dependent (i.e., trimer-specific) epitope, bound strongly while, in contrast, the non-NAb F105 against a CD4bs epitope reacted only minimally (Figure 4A). The F105 epitope is occluded on native trimers [10,21]. We have reported elsewhere that this MAb binds efficiently to BG505 gp120 monomers, and also to uncleaved (i.e., non-native) BG505 gp140 proteins [6,10]. Accordingly, the lack of F105 reactivity with the purified BG505 SOSIP.664 trimers is consistent with the absence of contaminant gp120 monomers of other non-native forms of Env such as uncleaved gp140 proteins. Of note is that the binding profiles obtained for the two MAbs were independent of the cell source of the trimers (Figure 4A).

The ELISA format involved trimer capture to the solid phase via the absorbed bNAb 2G12, followed by detection using biotin-labeled versions of the same PGT145 or F105 MAbs. As with the SPR system, an appropriate measure of structural authenticity for the trimers is strong PGT145 reactivity combined with low binding of F105. Again, irrespective of the trimer source, we found that PGT145 bound efficiently in the 2G12-capture ELISA whereas F105 was completely non-reactive (Figure 4B).

### Electron microscopy imaging of BG505 SOSIP.664 gp140 trimers produced from stable 293 T and CHO cell lines

**Figure 5:**
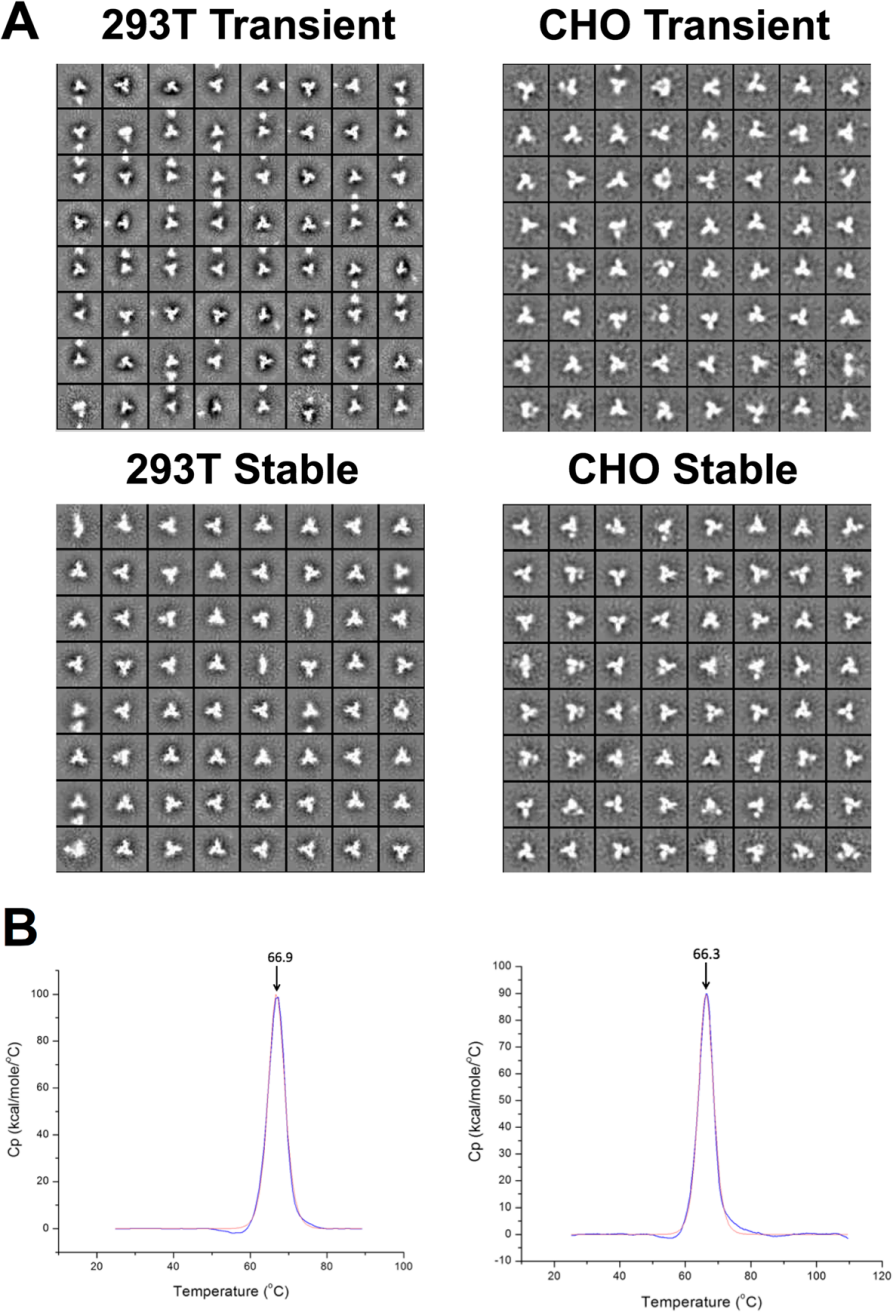
**Negative stain EM images and DSC traces for BG505 SOSIP.664 gp140 trimers produced by 293 T and CHO cells. (A)** Reference-free 2D class averages are shown for trimers from transiently transfected or Stable 293 T and CHO cell lines, as indicated. **(B)** Thermal denaturation profiles of BG505 SODSIP.664 trimers from the 293 T (left panel) and CHO stable cell lines (right panel).

The different preparations of purified trimers were viewed by negative-stain EM, and the reference-free 2D class averages were examined to determine their overall morphology. The trimers from both cell lines had the same consistently native-like appearance as those derived by transient transfection (Figure 5A) [10]. The percentages of native-like trimers varied in the range 81-100%, and were usually somewhat higher for 293 T cell-derived trimers (90-100%) than CHO (80-90%). However, we consider that native-like trimer percentages in this range (80-100%) are within experimental error for this method. Accordingly, we conclude that trimers from both the 293 T and CHO stable lines adopt a single native-like configuration that is indistinguishable, at this level of resolution, from the transient transfection products used to derive high-resolution cryo-EM and X-ray structures [13,14].

In a differential scanning calorimetry (DSC) assay, trimers produced in the Stable 293 T and CHO cell lines underwent a single, sharp thermal transition at 66.9**°**C and 66.3**°**C, respectively (Figure 5B). These values are comparable to those of 68.1**°**C for trimers produced by transient transfection of 293 T cells [10], and 67.9**°**C for ones made by transient transfection of 293S cells [22]. Overall, the DSC data further confirm the homogeneity of the different trimer preparations.

## Discussion

Here, we describe the production and properties of stable lines, derived on 293 T and CHO cell backgrounds, which express soluble, cleaved BG505 SOSIP.664 gp140 trimers. The 293 T and CHO lines each produced trimers at acceptable levels, with final yields after SEC purification of 10 mg and 12 mg per 1 × 10^9^ cells, respectively. The cell lines, of course, produce trimers continuously. Accordingly, we estimate that, under standard laboratory conditions, we can generate 20–40 mg of trimers from 10 fully confluent seeded hyperflasks, by re-feeding media and harvesting ~12-24 liters of supernatant over a 2–3 week period.

The purified BG505 SOSIP.664 gp140 trimers appear indistinguishable from ones produced by transient transfection of the same cell types, as judged by biochemical, antigenicity, EM and DSC analyses [10,22,23]. The cell line-derived trimers were fully cleaved, as determined by SDS-PAGE under reducing vs. non-reducing conditions (Figure 3C). The negative stain EM images and the antigenicity profiles are also completely consistent with efficient trimer cleavage, as uncleaved gp140 proteins have different antigenicity properties (i.e., F105-reactive, PGT145-nonreactive) and a distinctive, non-native appearance when viewed by EM [6]. The 293 T and CHO cell-derived trimers should therefore be suitable for both structural and immunogenicity studies. They may differ in the fine details of their glycosylation, given that the cells are of human and hamster origin, respectively. We have not, however, yet found any antigenic differences between 293 T- and CHO-cell derived trimers (with or without ELISA-enabling epitope tags), including with multiple bNAbs to glycan-dependent epitopes (Figure 4, and data not shown). Hence both cell lines are likely to be useful resources for trimer production. Of note is that BG505 SOSIP.664 gp140 trimers produced by transient transfection of 293 T and 293S cells were the reagents used to generate cryo-EM and x-ray structures, respectively, at ~5-6 Å resolution [13,14]. We are now making good progress towards a permanent cell line based on 293S cells, for additional x-ray diffraction studies.

We saw no indications that the cell line-derived BG505 SOSIP.664 trimers are degraded by proteases, including but not limited to ones that clip the V3 region to yield characteristic 70 kDa and 50 kDa fragments on reducing SDS-PAGE gels and Western blots (Figure 3D-F) [18,19]. In contrast, a monomeric gp120 (MN) and an uncleaved gp140 (CZA97.012) were highly vulnerable to proteolytic degradation, including but in the latter case not limited to V3 clipping. The uncleaved gp140 also formed aggregates even after the fraction corresponding to three gp120 and three gp41_ECTO_ subunits was first SEC-purified (Figure 3E and F). The resistance of the BG505 SOSIP.664 trimers to proteolytic degradation is at least partly because the BG505 V3 sequence is not particularly vulnerable to clipping, compared to other Env genotypes; we have, for example, seen no signs of V3 clipping when making monomeric BG505 gp120 proteins by transient transfection when, under the same conditions, MN gp120 is badly degraded (data not shown). However, there are also structural considerations; the SOSIP.664 trimers are compact entities in which potentially vulnerable scissile sites, particularly V3, are inaccessible to proteases [13,14]. We have also not observed V3 clipping or other signs of gp120 degradation with other SOSIP.664 trimers, including ones based on subtype B sequences (AC, JK and JPM, unpublished results). In contrast, V3 and other protease-sensitive regions are more exposed on both gp120 monomers and uncleaved gp140s. The latter invariably adopt splayed out, non-native shapes with three individual gp120 subunits dangling freely from a central gp41_ECTO_ 6-helix-bundle, conformations in which various sites, including V3, are exposed to protease attack [5,6]. In addition, uncleaved gp140 proteins form aggregates post-purification, which probably arises from the inappropriate exposure of hydrophobic sites within gp41_ECTO_. The native-like SOSIP.664 trimers do not form such aggregates.

The present cell lines were not made under GMP-conditions, but their creation serves as a proof of concept for the manufacture of GMP-compliant lines that could serve as the sources of BG505 SOSIP.664 gp140 trimers for human testing as vaccine antigens. CHO cell lines are commonly employed to make clinical grade recombinant proteins, and the use of 293 cell-based lines may also be feasible. We have recently re-designed the present pAM/C BG505 vector to facilitate the insertion of other *env* genes and thereby increase its flexibility as a device to produce SOSIP trimers based on a range of sequences. This vector system is also useful for making Furin-cleaved SOSIP trimers by transient transfection. We are presently assessing, in rabbits, its utility as a DNA vaccine vector for expressing cleaved SOSIP trimers *in vivo*.

## Conclusions

The stable CHO and 293 T cell lines yield non-degraded, native-like BG505 SOSIP.664 trimers of an appropriate quality for structural studies and animal immunogenicity experiments. The creation of these cell lines also represents a proof of concept for the production of these trimers under GMP conditions for human clinical trials.

## Methods

### Construct design

The BG505 SOSIP.664 gp140 *env* gene construct, based on the HIV-1 subtype A transmitted/founder virus BG505, has been described previously [10]. The gene was cloned into an expression plasmid containing a Flp Recombination Target (FRT) site linked to the hygromycin resistance gene, to permit Flp recombinase-mediated integration and facilitate the selection of a stable cell line expressing gp140 under the control of the human cytomegalovirus (CMV) immediate-early enhancer/promoter (Figure 1A). The *furin* protease gene was inserted into the same expression vector under the control of the EFI Alpha promoter. The resulting vector expressing both the gp140 and Furin proteins was termed pAM/C BG505. Large-scale plasmid preparations were prepared by transformation of DH5α-competent cells using a Qiagen™ EndoFree MaxiPrep kit according to the manufacturer’s instructions.

### Stable transfection of 293 T and CHO Flp-In™ cells

The Flp-In™ cells (293 T and CHO) were purchased from Invitrogen. These cells contain a single integrated FRT site and stably express the lacZ-Zeocin fusion gene. Parental 293 T Flp-In™ and CHO Flp-In™ cells were maintained in Dulbecco’s Modified Eagle’s Medium (DMEM) or Ham’s F-12 Medium, respectively, supplemented with 10% heat-inactivated FBS (Sigma, St. Louis, MO, USA), 200 U/ml penicillin/streptomycin, 2 mM L-glutamine, 20 mM HEPES, 0.1 mM non-essential amino acids, 1 mM sodium pyruvate (Gibco, Life Technologies, Carlsbad, CA, USA), and further supplemented with 100 μg/ml Zeocin (Invitrogen, Carlsbad, CA, USA).

Prior to transfection, 5 × 10^5^ cells were seeded into 6-well plates in 2 ml complete medium; they were ~70-80% confluent at the time of transfection. The pAM/C BG505 and pOG44 recombinase (Invitrogen, Carlsbad, CA, USA) expression vectors, the latter mediating integration into the FRT site of the Flp-In™ cells, were co-transfected using Lipofectamine 2000 (Invitrogen, Carlsbad, CA, USA) according to the manufacturer’s instructions. Briefly, 0.5 μg of the pAM/C BG505 and 4.5 μg of the pOG44 vectors (1:9 ratio) in Opti-MEM reduced serum medium (Gibco Life Technologies, Carlsbad, CA, USA) were mixed with 15 μl of Lipofectamine for 45 min at room temperature. The DNA/Lipofectamine complexes were then incubated with the cells for 6 h before addition of 2 ml fresh complete medium (without Zeocin). After culturing for a further 3 days, the cells were harvested and seeded (1 × 10^6^) into 100-mm Petri dishes (Falcon, San Jose, CA, USA) in the continued presence of hygromycin (150 μg/ml for 293 T cells, 500 μg/ml for CHO Flp-In™ cells). The selective medium was replenished every 3–4 days until discrete foci of hygromycin-resistant cells were evident after three weeks of selection.

### Clone selection

After the tissue culture medium was completely removed, a hydrophobic barrier was drawn around the hygromycin-resistant foci with a Pap Pen (Abcam, Cambridge, UK) before addition of 200 μl of 2.5% trypsin-EDTA (Invitrogen, Carlsbad, CA, USA). The released cells were harvested, seeded into 48-well plates and expanded in the presence of hygromycin. Cells were selected for further propagation once they were verified to be both hygromycin-resistant and to continually express HIV-1 Env, as assessed by dot blotting every 4–5 days (see below). Limiting dilution was performed according to recommendations in the manual ‘Corning Single Cell Cloning by Serial Dilution’. Briefly, 4000 cells were added to well A1 of a 96-well plate. Then, using a single channel pipettor, a 100 μl aliquot was transferred from the first well to well B and mixed by gentle pipetting. The 1:2 dilutions were then repeated down the entire column. Using an 8-channel micropipettor, an additional 100 μl of medium was added to each well in column 1. Then, using the same pipettor, 100 μl of medium was transferred from the wells in the first column (A1-H1) to those in the second column (A2-H2) and mixed. Using the same tips, the 1:2 dilutions were repeated across the entire plate so that all wells finally contained 100 μl of cell suspension. After culturing for ~2.5 weeks, cells were selected for subsequent expansion based on their rate of growth and their constitutive, high level expression of secreted trimers (as judged by ELISA; see below), or intracellular Env proteins (as determined by flow cytometry or fluorescent microscopy; see below). To assess cell viability changes with increasing passage number, we used the trypan blue exclusion test (Life Technologies, Carlsbad, CA, USA).

### Detecting Env expression by dot-blotting

Briefly, 2 μl of culture supernatant from cells under hygromycin selection were spotted onto nitrocellulose membrane and allowed to air-dry for 10 min. The membrane was blocked in 5% goat serum and incubated with MAb 2G12 (1:1000 dilution) for 1 h, followed by addition of biotinylated goat anti-human IgG (H + L) (1:2000 dilution) for 30 min. The membrane was then washed three times in Tris-buffered saline containing 0.05% Tween 20, incubated with VECTASTAIN® ABC Reagent (Vector Laboratories, Burlingame, CA, USA) for 20 min, and again washed three times in TBST before incubation with 3,3′,5,5′-Tetramethylbenzidine (TMB) substrate (Vector Laboratories, Burlingame, CA, USA) until the blue color was developed.

### Production of BG505 SOSIP.664 trimers and other Env proteins by transient transfection

Large-scale expression of BG505 SOSIP.664 trimers was performed using standard 293 T and CHO cells (see Results). The cells were seeded into hyperflasks (Corning Life Sciences, Union City, CA, USA) and transiently transfected with the pAM/C BG505 vector using linear polyethylenimine (PEI), as previously described [24]. Briefly, 600 μg of pAM/C BG505 was diluted in 60 ml of OPTI-MEM to which 3 ml of PEI (final concentration, 1 mg/ml, pH 7.5) was added drop-wise. After incubation for 20 min at room temperature, the DNA-PEI mix was diluted in 500 ml of complete medium and added to the cells. The supernatants were harvested 72 h after transfection and filtered prior to purification of the trimers.

Monomeric MN gp120 was expressed in 293 T cells by transient transfection using the MaxCyte electroporation transfection system, and purified by immunoaffinity chromatography using a CD4-IgG2 column coupled to CnBr-Sepharose (GE Healthcare, Fairfield, CT, USA). Briefly, transfection supernatants were passed over the antibody column. After washing the proteins were eluted with high salt, dialyzed into PBS and concentrated. The expression plasmid was provided by the IAVI NAC Reagent Repository.

Uncleaved CZA97.012 gp140 was expressed in 293 T cells by transient transfection via the PEI-based method described above, using a plasmid provided by Bing Chen (Harvard University). The resulting gp140 proteins contain a C-terminal Foldon domain and a His-tag, and were isolated on a Ni^2+^-column via their His-tags as described elsewhere [20]. SEC was then used to purify the fraction corresponding to three gp120 and three gp41_ECTO_ subunits.

### Purification of BG505 SOSIP.664 gp140 trimers

Trimers produced by the permanent cell lines or by transient transfection were purified as described elsewhere, with some minor modifications [6,10,13,25]. Env proteins in culture supernatants were first enriched by affinity chromatography on a Sepharose 4B column to which 2G12 (Polymun Sciences, Klosterneuburg, Austria) had been attached by CNBr-activation (GE Healthcare, Fairfield, CT, USA). The column was washed with 15–20 column volumes of buffer (500 mM NaCl, 10 mM Tris, pH 8.0) before Env proteins were eluted with 3–5 column volumes of 3 M MgCl_2_. The eluted Env proteins were immediately buffer exchanged into 75 mM NaCl, 10 mM Tris, pH 8.0 and then concentrated using Vivaspin ultrafilters (100 kDa MWCO; GE Healthcare, Fairfield, CT, USA). Trimers were separated from dimers and monomers by SEC on a Superdex HiLoad column (GE Healthcare, Fairfield, CT, USA). The trimer-containing fractions were pooled and concentrated. Protein concentrations were determined using a bicinchonic acid-based assay (BCA assay; Thermo Scientific, Waltham, MA, USA).

### SDS-page, blue native (BN)-page and western blotting

Env proteins were detected by SDS-PAGE, BN-PAGE and Western blotting, as previously described [7,25]. Briefly, Western blotting was performed after transfer of proteins onto a nitrocellulose membrane (Invitrogen, Carlsbad, CA, USA) at 32–40 V for 1–1.5 h. The membranes were blocked with 10% goat serum for 1 h at room temperature and incubated overnight with a 1:2000 dilution of the anti-gp120 MAb ARP 3119 (obtained from the NIBSC Reagent Repository), followed by a 1:5000 dilution of horseradish peroxidase-conjugated goat anti-mouse IgG (H + L) and the Western Lightning plus-ECL Enhanced Luminol Reagent (Perkin Elmer, Waltham, MA, USA). Alternatively, the membranes were probed with a HIV-Ig pool derived from individuals infected with viruses from subtypes A, B and C. The subtype A serum was obtained from Stephanie Rainwater (The Fred Hutchinson Cancer Research Center), the subtype C serum from Zdenek Hel (University of Alabama, Birmingham), and the subtype B serum from the AIDS Research and Reference Reagent Program (ARRRP) (product #3957, lot 110180). Each individual serum was present at a 1:3000 dilution in 10 ml of TBS + 0.05% Tween-20. The detection antibody was a 1:3000 dilution of biotin-conjugated goat anti-human IgG, followed by an Avidin-Biotin-HRP complex and a peroxidase substrate kit (all from Vector Laboratories, Burlingame, CA).

### Detecting intracellular Env expression by flow cytometry or fluorescent microscopy

We detected intracellular Env expression by using Fluorescein Isothiocyanate (FITC)-conjugated 2G12. To make FITC-2G12, the MAb was equilibrated into a sodium carbonate reaction buffer at pH 9.5 before FITC was added at a 10-fold molar excess (over IgG) for 30 min with gentle shaking. The mixture was fractionated using a Sephadex G-25 column with PBS as the eluent. The FITC-conjugated IgG concentration in the fractions (each 0.5 ml) was determined by measuring absorbance at 280 and 493 nm. The fluorescein/IgG ratio in the final pool of FITC-2G12 was calculated using the absorbance at 493 nm and molecular weights of 73 and 150 kDa for the respective components.

Stable transfectants or parental 293 T and CHO Flp-In™ cells were cultured for 6 h in the absence or presence of Brefeldin A (1 μg/ml; GolgiPlug™ from BD Biosciences, San Jose, CA, USA), which blocks the exit of excretory proteins from the endoplasmic reticulum. The cells were harvested after a brief treatment with trypsin-EDTA solution (2.5% trypsin/2.21 mM EDTA; Corning Life Sciences, Union City, CA, USA) and washed once in complete medium and twice in PBS (centrifugation at 300 g). To prevent non-specific binding of the detection MAb (i.e., FITC-2G12) to surface antigens, the cells were re-suspended in 100 μl of FACS buffer (PBS plus 2% FBS) and incubated with the “FcR block” reagent (Miltenyi Biotec; 5 μl/test) for 20 min at 4°C before addition of 250 μl of BD Fixation buffer (BD Biosciences, San Jose, CA, USA) for 20 min at 4°C. Following fixation, the cells were washed twice in 2 ml of Perm/Wash™ buffer (BD Biosciences, San Jose, CA, USA). To prevent non-specific MAb binding to intracellular antigens, the cells were further blocked with human AB serum (Sigma, St. Louis, MO, USA; 5 μl in 100 μl Perm/Wash™ buffer) for 20 min at 4°C, before incubation with FITC-2G12 (20 μg/ml) for a further 20 min at 4°C. Finally, the cells were washed three times in 2 ml Perm/Wash™ buffer and acquired using an LSR II FACS machine.

Alternatively, the 293 T and CHO cell clones were grown in an 8-well chamber slide (Thermo Scientific, Waltham, MA, USA) and treated with GolgiPlug™ (i.e., Brefeldin A) for 6 h. The cells were then fixed with 300 μl of acetone and methanol (1:1) at room temperature with gentle agitation for 10 min, washed five times in PBS, stained with FITC-2G12 (1:1000) for 1 h with agitation, and again washed five times in PBS. After counterstaining for 5 min with DAPI Nucleic Acid Stain (1:5000 dilution, Molecular Probes, Eugene, OR, USA), the culture chamber was removed using a slide separator and the slide mounted. Images were captured via a fluorescent microscope and digital camera (DMI6000B; Leica, Solms, Germany).

### Detecting BG505 SOSIP.664 gp140 trimers by ELISA

The ELISA to quantify the production of BG505 SOSIP.664 trimers by the cell lines was based on capturing Env proteins to the solid phase via MAb 2G12, and detecting trimers via biotin-labeled PGT145, a bNAb to a quaternary (i.e., trimer-dependent) epitope [21]. We also used biotin-labeled MAb F105 to probe for the production of undesired forms of Env, as the CD4-binding site (CD4bs) epitope for this non-neutralizing antibody (non-NAb) is inaccessible on native trimers but available on gp120 monomers and uncleaved gp140s [6,10]. MAb 2G12 was provided by Hermann Katinger through the ARRRP; MAbs PGT145 and F105 were provided by the IAVI Reagent Repository via Steven Fling.

For biotin conjugation, we used an EZ-Link Micro Sulfo-NHS-Biotinylation Kit (Thermo Scientific, Waltham, MA, USA). PGT145 or F105 (200 μg) was passed through a Zebra™ Spin desalting column and incubated with Sulfo-NHS-Biotin solution (11 mM) for 1 h at room temperature. The mixture was passed through a desalting column to remove excess biotin. The collected flow-through solution contains biotinylated MAb (bio-PGT145 or bio-F105) and was stored at 4°C.

Trimer production in culture supernatants from 293 T and CHO cell lines was assessed as follows. Briefly, Maxisorp ELISA plates were coated with 2G12 at 1 μg/ml overnight at 4°C (100 μl/well). The wells were washed three times with PBS containing 0.05% Tween 20 (PBST), and then blocked with PBS containing 2% non-fat milk for 1 h at room temperature. Culture supernatants were serially diluted and added to the wells for 2 h at room temperature. After three washes with PBST, 100 μl of bio-PGT145 or bio-F105 (1 μg/ml) was added for 1 h, before washing 3 times with PBST and addition of poly-HRP streptavidin (Thermo Scientific, Waltham, MA, USA; 1:2500 dilution in PBS containing 2% non-fat milk and 10% sheep serum) for a further 1 h. After a final 3 washes in PBST, the colorimetric endpoint was generated using 1-STEP Ultra TMB-ELISA substrate (Thermo Scientific, Waltham, MA, USA). Absorbance was measured at 450 nm.

### Assessing BG505 SOSIP.664 gp140 trimer antigenicity by SPR

MAb binding to purified trimers at 25°C was detected by surface plasmon resonance (SPR) with a Biacore 3000 instrument (GE Healthcare, Fairfield, CT, USA), essentially using one of the methods described elsewhere [10]. Env-reactive MAbs were captured onto CM5 chips by immobilized anti-Fc Ab, and the binding of solution-phase trimers was recorded. Affinity-purified goat anti-human IgG Fc (Bethyl Laboratories, Montgomery, TX, USA) was diluted to 50 μg/ml in sodium acetate (pH 4.5) and then amine-coupled to dextran, reaching levels ~10^4^ RU, in all four channels. MAbs were added at 1 μg/ml in running buffer (150 mM NaCl, 10 mM HEPES, pH 7.4, 3 mM EDTA plus 0.005% Tween 20) to three channels on each chip, at a flow rate of 5 μl/min, and captured to response level of 500–550 RU; a channel with only anti-Fc served as a control surface. Trimers, at 200 nM in running buffer, were injected at a flow rate of 50 μl/min. Association was recorded for 300 s and dissociation for 600 s. Background signals obtained from the control channel with trimer and from the test channel without trimer were both subtracted. The anti-human IgG-Fc surface was regenerated after each cycle by a single injection of 10 mM Glycine (pH 1.5) for 120 s at a flow rate of 30 μl/min.

### Visualizing BG505 SOSIP.664 gp140 trimers by negative stain electron microscopy

SEC-purified trimers were analyzed by negative stain EM as previously described [6,10]. A 3 μl aliquot containing ~0.03 mg/ml of the trimer was applied for 5 s onto a carbon-coated 400 Cu mesh grid that had been glow discharged at 20 mA for 30 s, then negatively stained with Uranyl formate for 30 s. Data were collected using a FEI Tecnai T12 or T20 electron microscope operating at 120 keV, with an electron dose of ~55e^-^/Å^2^ and magnifications of 52,000× or 29,000×. Images were acquired with a Tietz TemCam-F416 CMOS or Gatan US4000 camera using a nominal defocus range of 900 to 1300 nm.

### Differential scanning calorimetry

For thermal denaturation analysis, the 293 T and CHO cell-derived trimers were SEC-purified in phosphate buffered saline (PBS) before loading into the VP-differential scanning calorimetry (DSC) calorimeter (GE Healthcare, Fairfield, CT, USA). Thermal denaturation was conducted at a scan rate of 90°C/h. Buffer correction, normalization and baseline subtraction, and data analysis were conducted using the Origin 7.0 software.

## Abbreviations

BN-PAGE: Blue native-polyacrylamide gel electrophoresis; CD4bs: CD4-binding site; DSC: Differential scanning calorimetry; Env: Envelope glycoprotein; ELISA: Enzyme-linked immunosorbent assay; EM: Electron microscopy; FRT: Flp Recombination Target; GMP: Good manufacturing practice; HIV: Human immunodeficiency virus; HRP: Horseradish peroxidase; MAb: Monoclonal antibody; NAb: Neutralizing antibody; SEC: Size-exclusion chromatography; SPR: Surface plasmon resonance.

## Competing interest

PJK, RWS, ABW, IAW, JPM and AC are listed on a patent application relating to the general use of BG505 SOSIP.664 gp140 trimers. NPYC, KM, JPM, AM and AC will be listed on a patent filing relating to the cell lines described in this manuscript. The other authors have no competing interests to declare.

## Authors’ contributions

AC, AM and JPM conceived the study. NPYC, KM, MG, KR and PJK developed and analyzed the cell lines. TK developed and used the trimer ELISA. HJK, IAW and ABW performed EM and DSC analyses. JK and AC purified and characterized trimers. AY and PJK performed and analyzed SPR experiments. AM and AC designed the pAM/C vector. NPYC, KM, AC and JPM wrote the manuscript, with input from PJK, RWS, IAW and ABW. All authors read and approved the final manuscript.
